# Automatic Camera Calibration Using Active Displays of a Virtual Pattern

**DOI:** 10.3390/s17040685

**Published:** 2017-03-27

**Authors:** Lei Tan, Yaonan Wang, Hongshan Yu, Jiang Zhu

**Affiliations:** 1 College of Electrical and Information Engineering, Hunan University, Changsha 410082, China; wangyaonan@hnu.edu.cn; 2 National Engineering Laboratory for Robot Visual Perception and Control Technology, Hunan University, Changsha 410082, China; 3 College of Information Engineering, Xiangtan University, Yuhu District, Xiangtan 411105, China; jiang126@126.com

**Keywords:** camera calibration, 2D pattern, active display, lens distortion, closed-form solution, maximum likelihood estimation

## Abstract

Camera calibration plays a critical role in 3D computer vision tasks. The most commonly used calibration method utilizes a planar checkerboard and can be done nearly fully automatically. However, it requires the user to move either the camera or the checkerboard during the capture step. This manual operation is time consuming and makes the calibration results unstable. In order to solve the above problems caused by manual operation, this paper presents a full-automatic camera calibration method using a virtual pattern instead of a physical one. The virtual pattern is actively transformed and displayed on a screen so that the control points of the pattern can be uniformly observed in the camera view. The proposed method estimates the camera parameters from point correspondences between 2D image points and the virtual pattern. The camera and the screen are fixed during the whole process; therefore, the proposed method does not require any manual operations. Performance of the proposed method is evaluated through experiments on both synthetic and real data. Experimental results show that the proposed method can achieve stable results and its accuracy is comparable to the standard method by Zhang.

## 1. Introduction

Camera calibration is the first process for 3D computer vision which recovers metric information from 2D images. There are two types of approaches for calibration: photogrametric calibration uses both 2D information and knowledge of the scene such as coordinates of 3D points, shape of reference objects, direction of 3D lines, etc.; self-calibration does not require any knowledge but only 2D information. Generally speaking, the former approaches give more stable and accurate calibration results than the latter because using the knowledge reduces the number of parameters. The proposed method in this paper belongs to the photogrametric approaches.

The standard photogrametric calibration is Zhang’s method [1] which uses a 3D plane called a chessboard or checkerboard, even though many methods have been proposed which use perpendicular planes [2,3], circles [4,5], spheres [6,7], and vanishing points [8,9]. The merits of Zhang’s method are the ease of use and its extensibility. The requirement is only a camera and a paper on which a pattern is printed. Pattern images are captured by moving either the camera or the plane manually. Then, camera parameters are estimated by decomposing the homography between 3D points on the plane and their 2D projections on the image. The basic idea of Zhang’s method is not only for a single camera calibration, but also applicable to multiple camera calibration [10], projector-camera calibration [11], and depth sensor-camera calibration [12].

**Figure 1:**
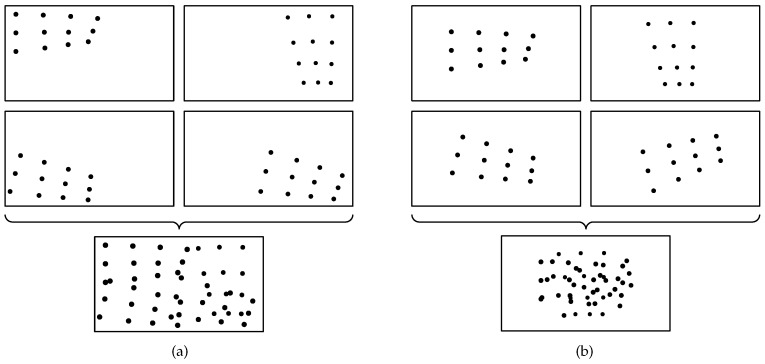
Distribution of Detected Points. (**a**) Detected points are distributed uniformly across the image; (**b**) Detected points are mainly located at the center part of the image.

Most parts of Zhang’s conventional method, such as checkerboard detection, can be automatically processed by software [13,14]. However, a manual part remains at the capture step. This part makes a calibration result unstable although it takes a lot of time. For stable calibration, many images under varied motions, generally ≥20 images, are required so that all detected points are distributed uniformly. Figure 1a shows an example in which all points from four images are scattered over the camera view. Otherwise, in a situation like Figure 1b, the conventional method does not give an accurate result for any trials.

To get well distributed points, robust methods are proposed for detecting partial occluded patterns [15,16,17]. By using those methods, if a part of the pattern is outside of the camera view, visible points including those near the image boundary are helpful for improving calibration accuracy. However, the manual part still exists.

This paper proposes a full-automatic calibration method to resolve the two problems caused by the manual operation: the time consuming problem and the point distribution problem. Instead of a physical pattern, the proposed method uses a virtual pattern which is transformed in the virtual world coordinates and projected on a fixed screen. The pattern on the screen is captured by a fixed camera, then, the proposed method performs calibration by using point correspondences between the virtual 3D points and their 2D projections. The virtual pattern can be actively displayed on the screen so that all points are uniformly distributed. Also, the camera and the screen are fixed during the whole process. Therefore, the proposed method can be stable and fully automatic.

This paper is organized as follows. Section 2 describes Zhang’s conventional method from basic equations. Although the derivation of Zhang’s method is widely known, it is highly related to the proposed method in Section 3. In Section 4, experimental results on synthetic and real images are provided and discussed. Finally, Section 5 gives the conclusions.

## 2. Conventional Method

**Figure 2:**
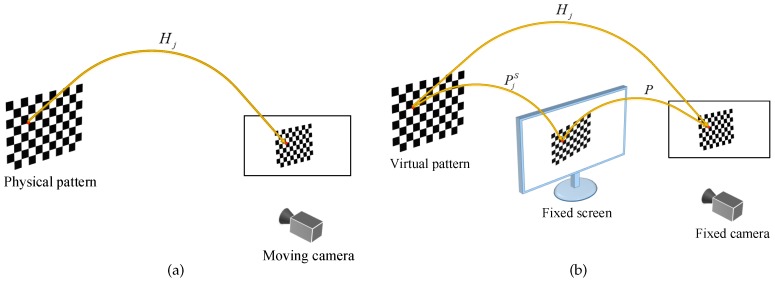
Overview of the conventional method and the proposed method. (**a**) The conventional method; (**b**) The proposed method.

Zhang’s conventional calibration method estimates the intrinsic and the extrinsic parameters of a camera from images of a physical planar pattern. Figure 2a shows an overview where the camera is moved by hand to take the pattern images.

### 2.1. Basic Equations

Assume that *n* 3D points are on a z=0 plane and the plane is shot by a pinhole model camera with *m* times. In a *j*-th shot (j≤m), the relation between a 3D point $X_{i}={\left[x_{i},y_{i},0\right]}^{T}$ (i≤n) and its 2D projection $m_{ij}={\left[u_{ij},v_{ij}\right]}^{T}$ can be expressed by

(1) $$\left[\begin{array}{c} m_{ij}\\ 1 \end{array}\right]\propto K\left[\begin{array}{cc} R_{j} & t_{j} \end{array}\right]\left[\begin{array}{c} X_{i}\\ 1 \end{array}\right]$$

where ∝ denotes equality up to scale, $R_{j}$ is a *j*-th 3×3 rotation matrix, $t_{j}$ is a *j*-th 3×1 translation vector, and *K* is a 3×3 upper triangular matrix given by

(2) $$K=\left[\begin{array}{ccc} f_{x} & s & u_{0}\\ 0 & f_{y} & v_{0}\\ 0 & 0 & 1 \end{array}\right]$$

with $\left[u_{0},v_{0}\right]$ the principal point, *s* the skewness, and $\left[f_{x},f_{y}\right]$ the focal length for *x* and *y* axis.

The third column of $R_{j}$ can be eliminated due to z=0. From Equation (1), then we have

(3) $$\left[\begin{array}{c} m_{ij}\\ 1 \end{array}\right]\propto K\left[\begin{array}{ccc} r_{j1} & r_{j2} & t_{j} \end{array}\right]\left[\begin{array}{c} x_{i}\\ 1 \end{array}\right]$$

where $x_{i}={\left[x_{i},y_{i}\right]}^{T}$, $r_{jk}$ denotes the the *k*-th column of $R_{j}$. Furthermore we can simplify this projection by using a 3×3 matrix

(4) $$H_{j}\propto K\left[\begin{array}{ccc} r_{j1} & r_{j2} & t_{j} \end{array}\right].$$

$H_{j}$, called a homography matrix, is given by at least four point correspondences $m_{ij}$ and $X_{i}$ [1]. Multiplying $K^{-1}$ from the left side of Equation (4) and using the orthogonality of $R_{j}$, we obtain two constraints for *K*:

(5) $$\begin{array}{c} \;h_{j1}^{T}Bh_{j2}=0, \end{array}$$

(6) $$\begin{array}{c} h_{j1}^{T}Bh_{j1}-h_{j2}^{T}Bh_{j2}=0 \end{array}$$

where $B\propto K^{-T}K^{-1}$, and $h_{jk}$ denotes the *k*-th column of $H_{j}$. *B* is a 3×3 symmetric matrix and has a six components. However, the degrees of freedom is five due to the scale ambiguity.

### 2.2. Estimating Parameters

Equations (5) and (6) are linear to *B*. Therefore, we can obtain *B* by solving

(7) Vvec(B)=0,

where *V* is a 2 m ×6 matrix and vec() is a vectorization operator. Note that the dimension of vec(B) is six. In a general case, where all the intrinsic parameters are unknown, m≥3 observations are required for getting a unique solution of vec(B). After getting *B*, *K* is extracted by decomposing *B*. More details on estimating the intrinsic parameters are described in [1] and [18].

Once *K* is known, $R_{j}$ and $t_{j}$ can be recovered as

(8) $$\begin{array}{c} R_{j}=\left[\begin{array}{ccc} \lambda K^{-1}h_{j1} & \lambda K^{-1}h_{j2} & r_{j1}\times r_{j2} \end{array}\right], \end{array}$$

(9) $$\begin{array}{c} t_{j}=\lambda K^{-1}h_{j3}\; \end{array}$$

with scale factor $\lambda =1/∥K^{-1}h_{j1}∥=1/∥K^{-1}h_{j2}∥$. Because of noisy data, $R_{j}=\left[r_{j1},r_{j2},r_{j3}\right]$ derived from the above equation does not generally satisfy the properties of a rotation matrix. The best rotation matrix from a general 3×3 matrix can be estimated through singular value decomposition [18].

### 2.3. Nonlinear Refinement

The estimated parameters above are not accurate because they are derived by linear methods based on the algebraic error without lens distortion. To refine the linear estimation, a nonlinear optimization is carried out by minimizing the re-projection error:

(10) $$\begin{array}{cc} \min_{K,R_{j},t_{j}\;\forall j\in m} & \sum_{j\forall m}\sum_{i\forall n}{∥m_{ij}-p\left(X_{i},K,R_{j},t_{j},d\right)∥}^{2}\\ s.t. & R_{j}^{T}R_{j}=I\;\forall j\in m \end{array}$$

where *I* is the 3×3 identity matrix, and *p* is a projective function with lens distortion parameter *d*.

## 3. Proposed Method

As shown in Figure 2b, the proposed method uses a virtual calibration pattern instead of a physical one. The virtual pattern is transformed by some pre-generated parameters and projected onto a screen, then, the pattern on the screen is captured by a fixed camera. For stable calibrations, the virtual pattern is actively displayed on the screen and these pre-generated parameters ensure that all 2D projections of the corner points are uniformly distributed in the camera coordinates. The proposed method estimates the intrinsic and the extrinsic parameters from correspondences between the virtual world points and their 2D projections.

In contrast to the conventional method, the proposed method does not require moving either the camera or the pattern. Since the camera and the screen are fixed during the whole process, the proposed method can be implemented as a fully automatic calibration software.

### 3.1. Basic Equations

Let $P=K\left[\begin{array}{cc} R & t \end{array}\right]$ be the projection from the screen to the camera and $P_{j}^{s}=K^{s}\left[\begin{array}{cc} R_{j}^{s} & t_{j}^{s} \end{array}\right]$ be the projection from the virtual pattern to the screen where $K^{s}$, $R_{j}^{s}$, and $t_{j}^{s}$ are the screen’s intrinsic and *j*-th extrinsic parameters, respectively.

Then, the projection between a virtual world space 3D point $X_{i}$ and a 2D image point $m_{ij}$ can be expressed by

(11) $$\left[\begin{array}{c} m_{ij}\\ 1 \end{array}\right]\propto \left[\begin{array}{cc} I & 0 \end{array}\right]\left[\begin{array}{c} P\\ 0^{T}\;1 \end{array}\right]\left[\begin{array}{c} P_{j}^{s}\\ 0^{T}\;1 \end{array}\right]\left[\begin{array}{c} X_{i}\\ 1 \end{array}\right]$$

where 0 is a 3×1 zero vector.

Let us consider the two projections separately. The first projection by $P_{j}^{s}$ can be rewritten by

(12) $$\begin{array}{cc} \left[\begin{array}{c} P_{j}^{s}\\ 0^{T}\;1 \end{array}\right]\left[\begin{array}{c} X_{i}\\ 1 \end{array}\right] & =\left[\begin{array}{cc} K^{s} & 0\\ 0^{T} & 1 \end{array}\right]\left[\begin{array}{cc} R_{j}^{s} & t_{j}^{s}\\ 0^{T} & 1 \end{array}\right]\left[\begin{array}{c} X_{i}\\ 1 \end{array}\right] \end{array}$$

(13) $$\begin{array}{cc} \; & =\left[\begin{array}{cc} K^{s} & 0\\ 0^{T} & 1 \end{array}\right]\left[\begin{array}{ccc} r_{j1}^{s} & r_{j2}^{s} & t_{j}^{s}\\ 0 & 0 & 1 \end{array}\right]\left[\begin{array}{c} x_{i}\\ 1 \end{array}\right] \end{array}$$

(14) $$\begin{array}{cc} \; & =\left[\begin{array}{c} H_{j}^{s}\\ 0\;1 \end{array}\right]\left[\begin{array}{c} x_{i}\\ 1 \end{array}\right] \end{array}$$

where $r_{jk}^{s}$ denotes the *k*-th column of $R_{j}^{s}$, and $H_{j}^{s}=K^{s}\left[\begin{array}{ccc} r_{j1}^{s} & r_{j2}^{s} & t_{j}^{s} \end{array}\right]$. $K^{s}$ is the screen’s intrinsic parameters which are preset in the calibration, and $R_{j}^{s}$ and $t_{j}^{s}$ are the extrinsic parameters of the screen at the j-th capture in the calibration. Since the virtual pattern is transformed by pre-generated parameters, $R_{j}^{s}$ and $t_{j}^{s}$ are actually known. Also the second projection by *P* can be rewritten by

(15) $$\begin{array}{cc} \left[\begin{array}{cc} I & 0 \end{array}\right]\left[\begin{array}{c} P\\ 0^{T}\;1 \end{array}\right] & =P \end{array}$$

(16) $$\begin{array}{cc} \; & =K\left[\begin{array}{cc} R & t \end{array}\right]. \end{array}$$

Letting $h_{jk}^{s}$ be the *k*-th column of $H_{j}^{s}$, and from Equations (14) and (16), we can write Equation (11) by using a 3×3 homography:

(17) $$\begin{array}{c} \left[\begin{array}{c} m_{ij}\\ 1 \end{array}\right]\propto H_{j}\left[\begin{array}{c} x_{i}\\ 1 \end{array}\right] \end{array}$$

where

(18) $$H_{j}\propto K\left[\begin{array}{ccc} Rh_{j1}^{s} & Rh_{j2}^{s} & Rh_{j3}^{s}+t \end{array}\right].$$

Similarly to the conventional method, given virtual world space 3D points and their 2D image projections, homography $H_{j}$ can be calculated using the same technique introduced in Zhang’s paper [1]. However, we cannot extract constraints from Equation (18) in the same way as Equations (5) and (6) since the form of $H_{j}$ is not identical. The proposed method uses the ratio constraints of the vector dot product instead of the orthogonality.

Multiplying $K^{-1}$ from the left side of Equation (18), we have three equations from the first and the second columns:

(19) $$\begin{array}{cc} \;∥K^{-1}h_{j1}{∥}^{2} & \propto ∥h_{j1}^{s}{∥}^{2} \end{array}$$

(20) $$\begin{array}{cc} \;∥K^{-1}h_{j2}{∥}^{2} & \propto ∥h_{j2}^{s}{∥}^{2} \end{array}$$

(21) $$\begin{array}{cc} {\left(K^{-1}h_{j1}\right)}^{T}\left(K^{-1}h_{j2}\right) & \propto h_{j1}^{sT}h_{j2}^{s} \end{array}$$

where $h_{jk}$ denotes the *k*-th column of $H_{j}$. If we take a ratio from any two of the above equations, we can obtain one constraints. For example, picking Equations (19) and (20), we have

(22) $$∥h_{j2}^{s}{∥}^{2}∥K^{-1}h_{j1}{∥}^{2}-∥h_{j1}^{s}{∥}^{2}{|K^{-1}h_{j2}∥}^{2}=0.$$

There are three possible combinations, but only two of them are linearly independent. Thus, we have two constraints by taking any two of them, e.g.,

(23) $$\begin{array}{cc} ∥h_{j2}^{s}{∥}^{2}h_{j1}^{T}Bh_{j1}-{∥h_{j1}^{s}∥}^{2}h_{j2}^{T}Bh_{j2} & =0, \end{array}$$

(24) $$\begin{array}{cc} \left(h_{j1}^{sT}h_{j2}^{s}\right)h_{j1}^{T}Bh_{j1}-{∥h_{j1}^{s}∥}^{2}h_{j2}^{T}Bh_{j1} & =0 \end{array}$$

with $B\propto K^{-T}K^{-1}$. Note that $h_{jk}$ and $h_{jk}^{s}$ are known but only *B* is unknown.

### 3.2. Estimating Parameters

As shown in Equations (23) and (24), we have two constraints from an $H_{j}$. Therefore, we can solve *B* and extract *K* in the same manner as the conventional method. On the other hand, a new approach is required for estimating the extrinsic parameters.

As soon as *K* is computed, a linear method can be employed to solve the extrinsic parameters. Stacking $K^{-1}H_{j}$ and $H_{j}^{s}$ for ∀j∈m horizontally, we have

(25) K−1H1⋯K−1Hm︸C=Rtμ1H1s0 1⋯μmHms0 1︸D

where $\mu_{j}=∥K^{-1}h_{j1}∥/∥h_{j1}^{s}∥$ is a scaling factor.

Then, Equation (25) can be linearly solved by

(26) $$\left[\begin{array}{cc} R & t \end{array}\right]=CD^{T}{\left(DD^{T}\right)}^{-1}.$$

### 3.3. Nonlinear Refinement

Nonlinear refinement must be applied to the linear estimation for more accuracy. The nonlinear optimization for the proposed method can be written by

(27) $$\begin{array}{cc} \min_{K,R,t} & \sum_{j\forall m}\sum_{i\forall n}{∥m_{ij}-p\left(X_{i},K^{s},R_{j}^{s},t_{j}^{s},K,R,t,d\right)∥}^{2}\\ s.t. & R^{T}R=I, \end{array}$$

where $p(X_{i},K^{s},R_{j}^{s},t_{j}^{s},K,R,t,d)$ is the projection of point $X_{i}$ onto the image, $d=[k_{1},k_{2}]$ denotes the lens distortion coefficients and all the screen parameters $K^{s}$, $R_{j}^{s}$, and $t_{j}^{s}$ are known. In our implementation, this optimization is also solved by using the Levenberg- Marquardt algorithm [19,20].

Distortion coefficients are estimated based on Zhang’s method [18] and included while minimizing Equation (27). For simplicity, only the first two coefficients of radial distortion $k_{1}$ and $k_{2}$ are considered, since the distortion function is mainly dominated by the radial components, especially the first term [2]. The relationship between the distortion-free pixel (x,y) and the distorted point $\left(x_{d},y_{d}\right)$ is presented by

(28) $$\begin{array}{c} x_{d}=x\left(1+k_{1}r^{2}+k_{2}r^{4}\right), \end{array}$$

(29) $$\begin{array}{c} y_{d}=y\left(1+k_{1}r^{2}+k_{2}r^{4}\right) \end{array}$$

where $r^{2}=x^{2}+y^{2}$. Readers can refer to [3] for more details on lens distortion model and how to compensate lens distortion.

### 3.4. Summary

The procedure of the proposed method is very similar to the conventional one and includes the following steps:

1. Place the camera in front of the screen and adjust its position and orientation;
2. Fix the camera when the whole camera view is covered by the screen and it contains as much part of the screen as possible;
3. Take a few images of the screen while the virtual checkerboard is being transformed and displayed;
4. Detect the corner points in the images;
5. Estimate focal length $f_{x}$ and $f_{y}$, principal point $\left[u_{0},v_{0}\right]$, skewness *s*, rotation matrix *R* and translation vector *t* using the closed-form solution as stated in Section 3.2;
6. Refine intrinsic and extrinsic parameters, including lens distortion coefficients, by nonlinear optimization as described in Section 3.3.

## 4. Experiments and Discussion

To demonstrate the validity and robustness of the proposed method, experiments on both synthetic data and real data have been conducted.

### 4.1. Experiment Setup

Before starting the calibration, the camera to be calibrated needs to be setup to ensure that the whole camera view is covered by a screen. To start with, the screen is placed within the working distance of the camera and the camera is looking straight to the screen. Ideally, using a screen with appropriate size and let the optical axis of a camera cross vertically with the screen at the center, the aforementioned condition should be satisfied. This setup may not work for a real camera, since its principal point is usually not at the center of the image. Also a real camera has lens distortion. Therefore, we still need to manually adjust the orientation and position of the camera, and fix the camera until its entire image is covered by the screen.

Then, a set of parameters about orientation and position are generated. They are used to transform the virtual pattern in the experiments. The orientation of the pattern is generated as follows: the pattern is parallel to the screen at first; a rotation axis is randomly chosen from a uniform sphere; the pattern is then rotated around that axis with an arbitrary angle θ between $40^{\circ }$ and $50^{\circ }$. The reason for choosing θ in that range is because it achieves the best performance according to the experimental results in [18]. The position of the pattern can be expressed by the 3D coordinate of its center point T=[x,y,z] in the screen’s coordinates. In order to generate appropriate position for the pattern, following scheme is adopted. The pattern and the screen are initially on the same plane, and the center of the pattern coincides with the center of the screen. The pattern is then moved along the positive direction of *Z* axis. When the projection of the pattern on the screen is about 1/4 size of the screen, the value of *z* is fixed. The value of *x* and *y* are determined by randomly choosing points on the plane Z=z, within the screen’s field of view. If given enough number (≥20) of patterns, all the 2D projections of the corner points should scatter all over the image and the uniform distribution is achieved.

### 4.2. Experiment on Synthetic Images

In the computer simulation, a simulated camera is created with the following intrinsic parameters: $f_{x}=1417$, $f_{y}=1420$, $u_{0}$ = 942, $v_{0}$ = 547, s=0, $k_{1}$ = −0.0806, $k_{2}$ = −0.0393. The screen which has 1920×1080 resolution can be described using ideal pinhole model with 2500 (in pixels) focal length, and the principal point is located at the center of the screen. The virtual checkerboard contains 16×10 = 160 corner points, and each square has 100 units per side. To investigate the performance of the proposed method regarding the noise level and the number of images of the calibration pattern, the following two experiments are designed and conducted. The method used for corner detection in the experiments is the method developed by Vezhnevets Vladimir, which is also integrated in OpenCV [21].

**Figure 3:**
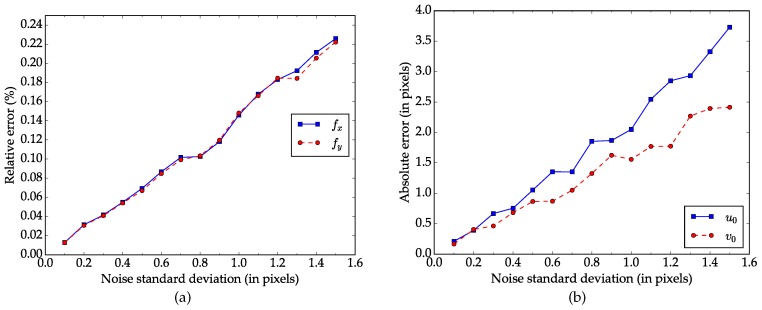
Errors regarding the noise level of the image points. (**a**) Relative error for focal length; (**b**) Absolute error for principal point.

**Performance regarding the noise level.** To start with, virtual patterns with 20 different orientations and positions are synthesized. Then noisy images are created by adding Gaussian noise with a mean of μ=0 and a standard deviation of σ to the projected image points. The noise level varies from σ=0.1 to σ=1.5. For each noise level, our method is tested with 100 independent trials and assessed by comparing the results with the ground truth. Figure 3a,b show the relative error for focal length and absolute error for principal point respectively. As we can see in Figure 3, the average errors increases as the the noise level rises and the relationship between them is almost linear. When the noise level increases to σ=0.5, which is larger than the normal noise in practical calibration [18], the relative errors in focal length $f_{x}$ and $f_{y}$ are less than 0.1%, and the absolute errors in principal point $u_{0}$ and $v_{0}$ are around 1 pixel.

**Figure 4:**
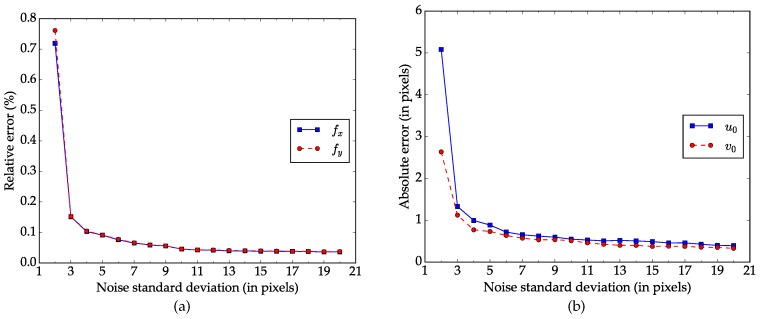
Errors regarding the number of the calibration pattern. (**a**) Relative error for focal length; (**b**) Absolute error for principal point.

**Performance regarding the number of images.** This experiment is designed to explore how the number of images of the calibration pattern impacts the performance of our method. Starting from two, we increase the number of images by one each time until it reaches twenty. For each number, Gaussian noise(μ=0, σ=0.5) is first added to the images, calibration is then conducted with these independent images for 100 times. The errors are calculated based on the calibration results and ground truth data as in the previous experiment. The mean values of the errors are shown in Figure 4. The errors decrease and tend to be stable as the number of image increases. Note that the errors decrease significantly when the number increases from 2 to 3.

### 4.3. Experiments on Real Images

**Figure 5:**
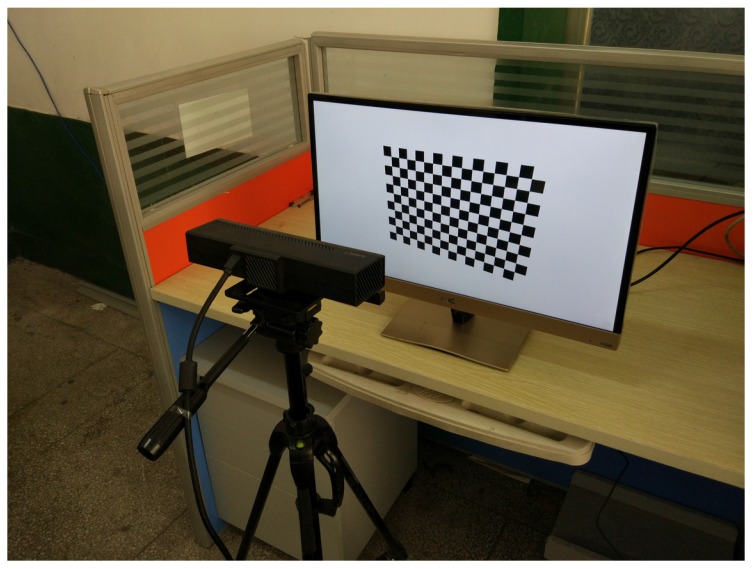
Setup of the real experiment.

**Figure 6:**
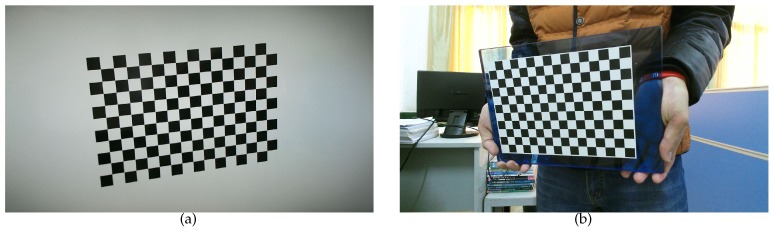
Two calibration images captured in real experiment. (**a**) Image of a virtual checkerboard shown on screen; (**b**) Image of a physical checkerboard.

To test our method on real images, we use a 24 inch LCD monitor to display the virtual pattern. Parameters of the screen and the virtual pattern are the same as in the computer simulation. The camera to be calibrated is the color camera of a Microsoft Kinect for Windows V2 sensor. As shown in Figure 5, the camera is fixed approximately 40 cm away from the screen using a tripod, looking straight to the screen, so that the whole camera view is covered by the screen. Ten independent trials are performed with images of 1920×1080 resolution. In each trial, virtual pattern is transformed using parameters randomly chosen from the synthetic data and shown on the monitor. Meanwhile, the screen is captured by a real camera and 20 different images are used in each calibration. Figure 6a shows sample images captured in this experiment. The images are collected automatically by computer program, and the screen and the camera are fixed during the whole process. We use the same method as in the synthetic experiments for corner detection.

In comparison, we also calibrated the real camera using a physical checkerboard. The pattern is printed by a high-quality printer and attached to a glass board with guaranteed flatness. It contains the same number of squares as the virtual pattern, and each square is 15 mm × 15 mm. The camera is fixed by a tripod, and images are collected while the checkerboard is being manually moved. A sample images used in this experiment is shown in Figure 6b. Ten independent trials are performed, with 20 images each time.

**Figure 7:**
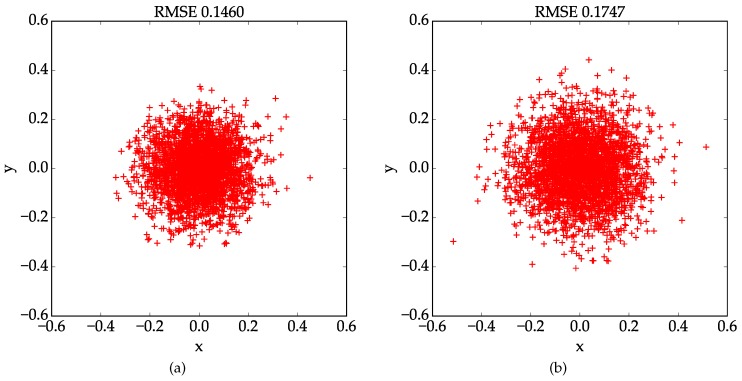
Scatter plots for the RMSE between the detected corner points and the re-projected ones with the estimated calibration parameters. (**a**) Localization errors by the proposed method; (**b**) Localization errors by the conventional method.

Explicit calibration experiments results are reported in Table 1 and Table 2. For the first 10 lines in the tables, each line shows the result obtained in an independent trial, which are the 6 camera parameters and the root mean square error( RMSE). Here, the RMSE is defined as the root mean square distance between every detected corner point and the re-projected one using the estimated parameters. The mean and standard deviation values of the estimated parameters are listed in the last two lines. As we can see in Table 1, results obtained using the proposed method are very consistent with each other and the standard deviations for all parameters are pretty small, which suggests that our method is very robust. Contrarily speaking, performance of the conventional results are not as stable as the proposed one. Since we don’t have ground truth data of the real world experiment, the camera parameters estimation result is evaluated based on re-projection error. With the proposed method and the conventional one, the mean value of the RMSE are 0.1855 and 0.2337 pixels, respectively. And the lowest RMSE, which is 0.1460, is achieved by the proposed method. We choose the best calibration results obtained by our method and the conventional method, and plot the localization errors of the control points in Figure 7. The results indicate that the proposed method outperforms the conventional one in terms of stability and accuracy in real world experiments.

### 4.4. Discussion

The above experiments show not only the practicality but also the advantage of the proposed method. In conventional calibration method, a key step is to capture images while manually moving a physical calibration pattern. Usually, this step takes as long as several minutes. In contrast, our method takes much less time to prepare calibration pattern and collect high quality data, and the whole procedure is done fully automatically within one minute.

**Figure 8:**
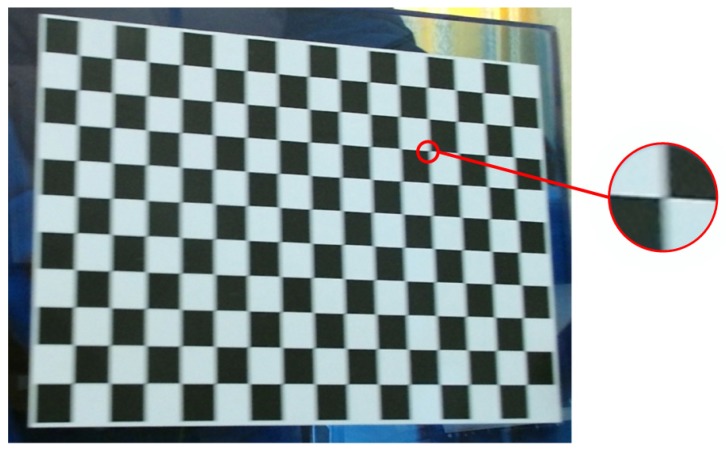
A blurry image captured in real experiment.

The use of virtual pattern affects the calibration result in the following aspects. First, virtual pattern is transformed by computer program so that all the control points are uniformly distributed in the image. Well distributed points usually lead to more stable and accurate calibration result. Second, since the screen is fixed in the calibration, image blur caused by motion can be eliminated, therefore, control points can be more precisely localized. Otherwise, in a blurry image which is taken by a moving camera like Figure 8, the observed feature location in the image may deviate from the actual feature location. Even though the checkerboard patten can be detected by some algorithms (e.g., OpenCV’s checkerboard detection algorithm [21]), uncertainty in the localizations of the control points yields incorrect correspondences which lead to performance degradation of the calibration.

However, the proposed method also shows some limitations. An essential requirement of this method is that the entire camera view has to be covered by a screen. In some cases, it is difficult to satisfy the above requirement. For a camera with large working distance or wide field of view, it is necessary to use a large size screen, e.g., flat screen TV, to cover the entire image of the camera. However, screen size cannot be increased without limitation, our method may not be applicable if the camera has very large working distance or very wide field of view. The proposed method also does not work in some certain applications, such as high precision visual measurement, where the camera to be calibrated has very short working distance or very high resolution. In this case, the resolution of the camera is usually higher than that of the screen. Hence the image of a screen is discretized, and corner point detection and localization can be a problem. Although the effect of discretization can be reduced by using high resolution screen, it still affects the accuracy of calibration unless it is completely eliminated.

## 5. Conclusions

The conventional calibration technique using a 2D planar object is widely used due to its ease of use. Although many efforts have been focused on making the whole calibration procedure as automatic as possible, there is still a manual part at the capture step which takes a lot of time and makes the result unstable. In this paper, we proposed a full-automatic method for camera calibration to resolve the issues brought about by manual operations. Different from the conventional method, we use a virtual pattern which is transformed in the virtual world coordinates and projected on a fixed screen. The pattern shown on the screen is then captured by a fixed camera. Calibration is performed by using point correspondences between the virtual 3D points and their 2D projections, and the solution to camera parameters estimation is very similar to the conventional method.

Owing to the use of virtual pattern, there is no need to manually adjust the position and orientation of the checkerboard during calibration. Moreover, the virtual pattern can be actively displayed on the screen so that all corner points are uniformly distributed. Once the camera and the screen are set up, they are fixed during the whole calibration process. Thus, the proposed method can be fully automatic and the problems caused by manual operation are resolved without loss of usability. Experimental results show that our method is more robust and accurate than the conventional method.

## Figures and Tables

**Table 1:** Calibration result for real images using the proposed method.

	$f_{x}$	$f_{y}$	$u_{0}$	$v_{0}$	$k_{1}$	$k_{2}$	RMSE
**Trial 1**	1050.2120	1045.9939	957.1198	519.4579	0.0448	−0.0468	0.1502
**Trial 2**	1052.1709	1047.9542	957.1122	519.7247	0.0456	−0.0494	0.2021
**Trial 3**	1048.5039	1044.3648	956.7213	519.2291	0.0442	−0.0462	0.2061
**Trial 4**	1051.0054	1046.8187	956.8339	519.2194	0.0455	−0.0486	0.1756
**Trial 5**	1050.8918	1046.7329	956.8582	519.4178	0.0460	−0.0498	0.1944
**Trial 6**	1051.2977	1047.1457	956.8358	519.4241	0.0454	−0.0481	**0.1460**
**Trial 7**	1050.4691	1046.3180	956.5077	519.6354	0.0446	−0.0467	0.1699
**Trial 8**	1052.8643	1048.7560	956.4323	519.5267	0.0452	−0.0473	0.2077
**Trial 9**	1051.0076	1046.8497	956.9606	519.6325	0.0461	−0.0489	0.1952
**Trial 10**	1049.4690	1045.3789	956.4628	519.2602	0.0460	−0.0494	0.2076
**Mean**	1050.7892	1046.6313	956.7845	519.4528	0.0453	−0.0481	0.1855
**Deviation**	1.2463	1.2397	0.2515	0.1791	0.0006	0.0013	0.0236

**Table 2:** Calibration result for real images using the conventional method.

	$f_{x}$	$f_{y}$	$u_{0}$	$v_{0}$	$k_{1}$	$k_{2}$	RMSE
**Trial 1**	1048.0347	1044.0247	956.8945	519.3556	0.0438	−0.0464	0.2595
**Trial 2**	1047.9891	1043.7756	956.6410	519.7846	0.0458	−0.0485	0.2153
**Trial 3**	1051.6414	1047.3967	957.3807	519.6939	0.0458	−0.0486	0.2029
**Trial 4**	1052.1863	1048.0365	957.2387	519.3653	0.0454	−0.0470	0.2948
**Trial 5**	1050.3806	1046.1871	956.9527	519.0593	0.0446	−0.0452	0.2469
**Trial 6**	1049.6929	1045.5486	956.8276	519.5737	0.0451	−0.0475	0.2210
**Trial 7**	1048.9989	1044.8639	956.8082	519.3750	0.0449	−0.0465	**0.1747**
**Trial 8**	1050.1785	1046.0461	956.7260	519.5743	0.0439	−0.0457	0.2672
**Trial 9**	1050.3922	1046.2240	956.5963	519.5850	0.0437	−0.0445	0.1787
**Trial 10**	1051.6436	1047.4263	956.8238	519.8481	0.0450	−0.0459	0.2757
**Mean**	1050.1138	1045.9530	956.8889	519.5215	0.0448	−0.0466	0.2337
**Deviation**	1.4674	1.4353	0.2487	0.2362	0.0008	0.0014	0.0414
